# Nasal and extra nasal MRSA colonization in hemodialysis patients of north-west of Iran

**DOI:** 10.1186/s13104-019-4298-9

**Published:** 2019-05-10

**Authors:** Fatemeh Ravanbakhsh Ghavghani, Leila Rahbarnia, Behrooz Naghili, Alireza Dehnad, Ahad Bazmani, Mojtaba Varshochi, Mohammad Hossein Ghaffari Agdam

**Affiliations:** 1 Infectious and Tropical Diseases Research Center, Tabriz University of Medical Sciences, P.O.Box; 5163639888, Tabriz, Iran; 2 Higher Education Institute of Rab-Rashid, Tabriz, Iran, Tabriz, Iran; 3 Biotechnology Department, East Azerbaijan Research and Education Canter Agricultural and Natural Resources, AREEO, Tabriz, Iran; 4 Drug Applied Research Center, Tabriz University of Medical Sciences, Tabriz, Iran

**Keywords:** *S. aureus*, MRSA colonization, Hemodialysis patients

## Abstract

**Objectives:**

Methicillin resistant *Staphylococcus (S.) aureus* colonization is one of the main causes of serious infections in hemodialysis patients. This cross-sectional study was performed to examine prevalence of MRSA colonization and evaluation of risk factors in hemodialysis patients. A total of 560 swab samples from nasal, the skin around catheter and throat were collected from 231 hemodialysis patients in Tabriz. The standard biochemical tests were used for identification of *S. aureus* isolates. Antimicrobial susceptibility profile was determined against 11 antibiotics by the disk diffusion method. Phenotypic test of *S. aureus* was performed using novobiocin 30 μg/disc, and methicillin sensitivity test was performed by cefoxitin 30 μg/disc.

**Results:**

Overall, 50.65% (118/231) hemodialysis patients were positive for *S. aureus* which 34.93% (80/231) of patients were MRSA carriage. The MRSA colonization in patients with a catheter (44.06%) was more than individuals utilizing a fistula (24.57%, *p* = 0.030). Among sampling sites, the highest MRSA was related to nasal samples (30.70%, *p* < 0.00001). Extra nasal colonization of *S. aureus* was observed in 12.71% patients. The highest rates of resistance were observed against ampicillin (93.98%) and the highest sensitivity was against linezolid antibiotic (5.42%). These findings highlight the necessity of prophylaxis against *S. aureus* in individuals under dialysis.

## Introduction

The prevalence of bacterial infections is one of the main reasons of mortality in hemodialysis patients. In fact, the weakened immune system and nature of dialysis devices predispose hemodialysis patients for infection. Based on recent reports, *Staphylococcus (S.) aureus* is the most prevalent bacterial infection in hemodialysis individuals ranging from minor skin infections to serious infections including bacteremia, osteomyelitis, necrotizing pneumonia, infective endocarditis, and toxic shock syndrome (TSS) [1]. It is capable of colonization in the different sites of body such axillary, perineal and nasal (nasal carriage of *S. aureus*) [2, 3].

The nasal carriage of *S. aureus* is not often the main cause of infection however it can act as a reservoir for subsequent infections in individuals colonized with this pathogen [4, 5].

At present, the emergence of antibiotic resistant *S. aureus* strains known as methicillin-resistant *S. aureus* (MRSA) has become a serious menace in hospital and community settings. The MRSA infections are mainly associated with increasing morbidity and mortality and length of hospital stay especially in individuals with cancer, HIV, diabetes, rheumatoid arthritis and patients undergoing dialysis [1].

The origin of *S. aureus* infections in dialysis patients is mostly related to *S. aureus* nasal carriage. This microorganism is transmitted from the nasal reservoir to the hands and skin, and then to the access. So, the prevention of colonization of *S. aureus* can be an effective method to infection control in hemodialysis patients. This study was aimed to examine prevalence of MRSA colonization in hemodialysis patients as a main cause of infection and mortality in hemodialysis population.

## Main text

### Methods

#### Sample collection

The swab samples were collected from hemodialysis patients referred to the Imam Reza hospital of Tabriz, Iran from July 6, 2018 to August 6, 2018. The samples were obtained from three different sites including nasal, throat and skin around the catheter. Patients’ clinical data including gender, age and clinical conditions were recorded before samples collection. The relationship between *S. aureus* carriage and clinical conditions of hemodialysis patients such as hypertension, diabetes mellitus, glomerulonephritis, lupus, familial Mediterranean fever, polycystic kidney, cardiovascular, pulmonary infections and length of hospitalization, the effect of risk factors such smoking was also examined. The procedures followed were according to the ethical standards of the committee on human experimentation of our institution.

#### Bacteria identification

The samples were cultured in selective media (Manitol Salt agar and blood agar media) and incubated overnight at 37 °C. The *S. aureus* isolates were identified based on conventional microbiological and biochemical tests (Gram-staining, colony morphology, coagulase test (coagulase was positive for *S. aureus*) and catalase test [6].

#### Antimicrobial susceptibility testing

The susceptibility pattern of isolates to various antibiotics was determined by Kirby Bauer disk diffusion testing based on the Clinical Laboratory Standards Institute (CLSI) guidelines [7]. Antibiotic disks used were as follow: oxacillin (1 μg), mupirocin (10 μg), rifampicin (5 μg), erythromycin (15 μg), clindamycin (2 μg), minocycline (30 μg), linezolid (30 μg), cefazolin (30 μg), and trimethoprim-sulfamethoxsazole (1.25/23.75 µg), ampicillin (5 μg), cefoxitin (30 μg), novobiocin (5 μg). Muller–Hinton agar plats were used for susceptibility tests and the bacterial concentrations for inoculation of plates were equal to 0.5 McFarland. The disks were incubated with the inoculated plates at 37 °C overnight. Based on CLSI guideline, *S. aureus* ATCC 25923 (methicillin susceptible) and *S aureus* ATCC 33591 (methicillin resistant) were used as positive control and ultrapure water was as negative control.

#### Statistical analysis

Statistical analysis was carried out by the SPSS version 16. Demographic and clinical variables were compared by Chi square test (*p* < 0.05).

### Results

#### Bacterial identification

A total of 560 samples from nasal, throat and skin around the catheter were obtained from 231 hemodialysis patients. A total of 166 *S. aureus* isolates (29.64%) were isolated from 560 samples which 62.04% (103/166) of *S. aureus* isolates were MRSA. Totally, from 231 patients, 118 individuals (50.65%) were identified as *S. aureus* carriage which 67.79% (80/118) of them were MRSA carriers. There was no significant difference in the prevalence of MRSA among hemodialysis patients with history of past illness. Demographic data of patients including gender, age, risk factors, illness history and the types of collected clinical specimens were summarized in Table 1. According to statistical analysis results, the frequency of MRSA is independent on gender (*p* = 0.302). But, it was dependent on sampling site so that prevalence of MRSA was significantly higher in nasal samples (30.70%) (*p* < 0.00001). *S. aureus* colonization in the absence of nasal colonization was observed in 12.71% (15/118) patients. Based on results, frequency of MRSA colonization was dependent on type of vascular access used in hemodialysis patients (*p* = 0.032) so that 44.06% of MRSA carriers were related to hemodialysis patients with a catheter.

**Table 1:** Demographic data of hemodialysis patients admitted to Imam Reza hospital of Tabriz

Study group (n = 231)	MRSA	MSSA	*p* value
Gender			
Female (n = 86)	35	15	0.302
Male (n = 145)	45	23	
Age			
17–30 (n = 21)	2	7	0.0522
31–40 (n = 15)	6	2	0.35238
41–50 (n = 27)	10	8	0.846755
51–60 (n = 56)	20	11	0.891901
> 60 (n = 110)	37	15	0.832589
Samples (n = 560)			
Nasal (s = 228)	70	37	< 0.00001
Trout (s = 225)	15	11	
Around catheter (s = 114)	18	14	
Vascular access for dialysis			
Catheter (n = 116)	52	16	0.030, < 0.05
Fistula (n = 115)	29	21	
Illness history			
Hypertension (n = 129)	50	20	0.299
Diabetes mellitus (n = 102)	31	18	0.399
Glomerulonephritis (n = 10)	4	2	0.95
Polycystic kidney (n = 11)	4	3	0.336
Pulmonary infections (n = 6)	2	1	0.962
Cardiovascular (n = 22)	9	3	0.555
Familial Mediterranean fever (n = 1)	1	1	0.761
Lupus (n = 2)	2	0	0.259
Length of hospitalization (27)	11	6	0.627
Smoking (17)	4	5	0.118
Antibiotic consumption (n = 34)	14	5	0.562

n, number of patients; s, number of samples

#### Antimicrobial susceptibility

**Fig. 1:**
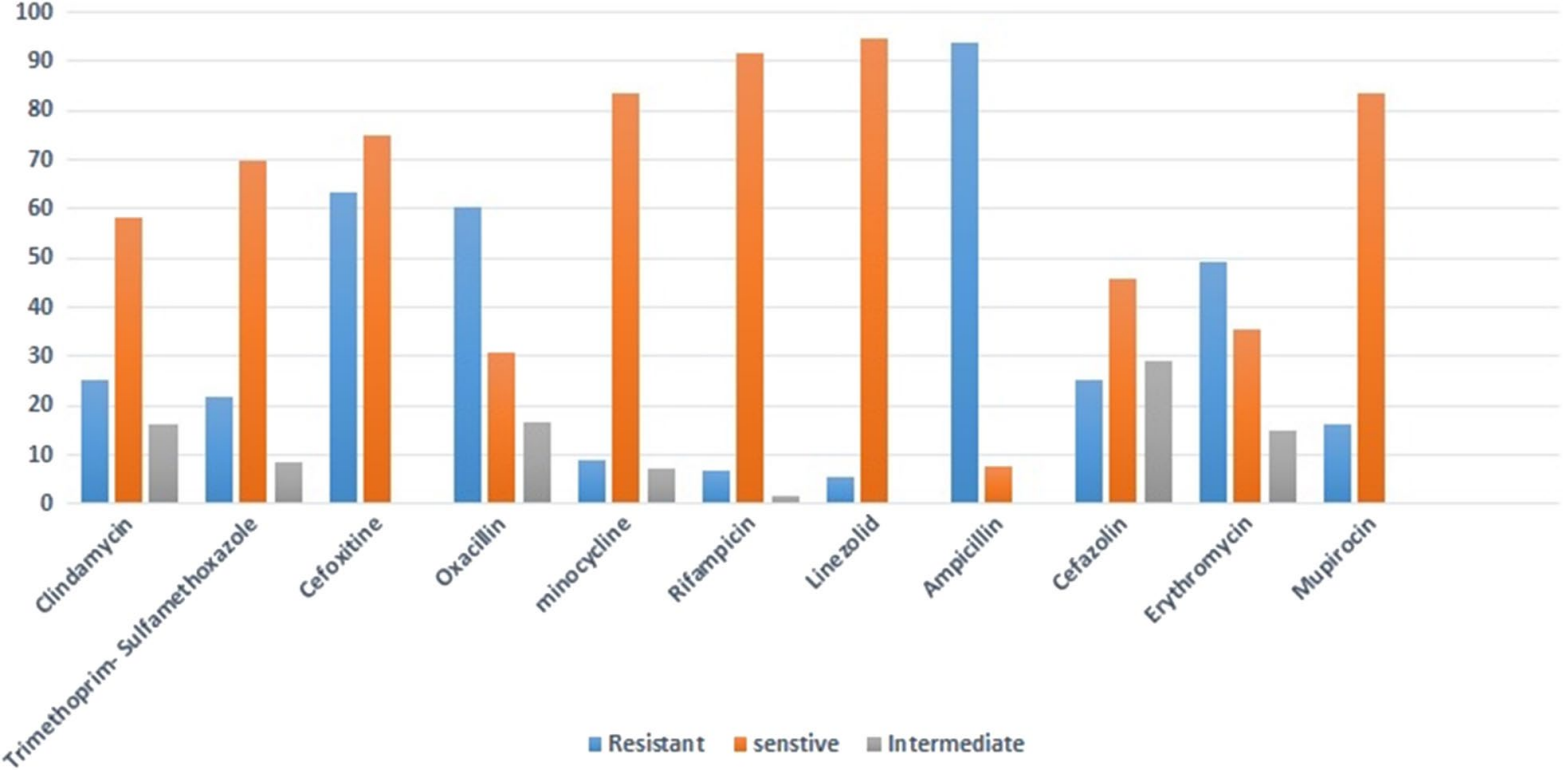
Results of antibiotic susceptibility test of *S. aureus* strains isolated from hemodialysis patients

Antibiotic resistance profile to 11 antibiotics was determined on 166 *S. aureus* isolates by the disc diffusion agar. The highest antibiotic resistance rate was observed against Ampicillin (93.98%) and the highest sensitivity was against linezolid antibiotic disk (5.42%). However, based on CLSI guideline, intermediate sensitivity values to linezolid was not reported. Based on results of resistance to cefoxitin, 34.93% (80/231) of patients were MRSA carriage. Details of results of the disc diffusion test are shown in Fig. 1. Out of the 166 *S. aureus* isolates, 90 (54.22%) isolates including 58 (34.94%) nasal, 16 (9.64%) catheter, 16 (9.64%) throat showed multi-drug resistant property (MDR) based on resistance to three or more classes of antibiotic [8] and 2 isolates (1.2%) were resistant to all of antibiotics.

### Discussion

At present, increasing prevalence of MRSA infections has become a serious issue in clinical settings due to MRSAs are resistant to most of antibiotic classes especially β-lactams [9]. According to researches done, there is a direct relationship between *S. aureus* colonization and incidence of infection [10, 11]. In the present study, *S. aureus* colonization was detected in 50.65% of hemodialysis patients that 67.79% of *S. aureus* isolates were found as MRSA and totally 34.93% of patients were identified as MRSA carriage. In a study conducted in north of Iran, 36.9% of hemodialysis patients were found as *S aureus* carriage which MRSA colonization was detected in 74.2% of *S. aureus* carriers [12]. But, in a study carried out on hemodialysis patients in South of Iran, only 19.67% of individuals, were carriers of S. aureus and 6.56% were MRSA carriage [13]. The rate of *S. aureus* colonization at nasal, throat and catheter around was documented in 30.78%, 6.66% and 15.78% of hemodialysis patients while in one report offered from north of Iran, 36.9% of hemodialysis patients were *S. aureus* nasal carriage [12].

Also, in another study carried out by Devraj et al. *S. aureus* colonization in throat were observed in 11.4% of hemodialysis patients [14].

In our study, 44.06% of MRSA carriers were related to hemodialysis patients using from a catheter. In this respect, researches showed that the possibility of infection in patients with catheter is two- to threefold higher than individuals with a fistula or graft [15].

In the present study, 25% of *S. aureus* isolates were resistant to Clindamycin, consistent with results obtained from south of IRAN [13]. Also, 6.63% of *S. aureus* isolates were resistant to Rifampicin similar with study carried out in Mashhad, Iran [16].

In this study, there was no significant difference in the prevalence of MRSA colonization among hemodialysis patients with past illness history. These results were consistent with the similar results regarding lack of relationship between *S. aureus* colonization and clinical conditions of hemodialysis patients such as diabetes and Hypertension [12, 14]. In the present study, MRSA colonization was independent of age consistent with similar studies [17, 18].

Although, *S. aureus* colonization is often detected in nasal, there are evidences regarding extra nasal *S. aureus* colonization in patients [19, 20]. Our results are indicating incidence of 12.711% extranasal *S. aureus* colonization without nasal colonization in patients that is similar to results of Zahed et al. conducted in Tehran, Iran regarding incidence of 12% extranasal *S. aureus* colonization in hemodialysis patients [21].

### Conclusion

Our results were indicating incidence of nasal and extranasal MRSA colonization in hemodialysis patients in the study region. These findings highlight the necessity of prophylaxis against *S. aureus* in individuals under dialysis.

## Limitations

Since, the conventional biochemical tests are not definitive and can misidentify *S. aureus* isolates so PCR method can be used for confirmation of *S. aureus* and MRSA isolates.

## Data Availability

All data generated or analyzed during this study are included in this published article.

## Abbreviations

MRSA: methicillin resistant *Staphylococcus (S.) aureus*
TSS: toxic shock syndrome
MDR: multi-drug resistant
